# Topical JAK inhibitor for refractory skin inflammation in STAT1 GOF

**DOI:** 10.70962/jhi.20250049

**Published:** 2025-06-16

**Authors:** Valentina Guarnieri, Tiphaine Arlabosse, Claire Booth, Gabriela Petrof

**Affiliations:** 1 Department of Immunology and Gene Therapy, Great Ormond Street Hospital for Children NHS Foundation Trust, London, UK; 2 Department of Health Sciences, University of Florence, Florence, Italy; 3 UCL Great Ormond Street Institute of Child Heath, London, UK; 4 Department of Dermatology, Great Ormond Street Hospital for Children NHS Foundation Trust, London, UK

## Abstract

Topical ruxolitinib led to marked improvement in refractory facial dermatitis in a 10-year-old patient with STAT1 gain-of-function. These findings support localized inhibition of dysregulated JAK–STAT signaling as a well-tolerated and effective treatment for cutaneous manifestations of STAT1 gain-of-function disease.

## Teaser

Topical ruxolitinib induced rapid and sustained improvement of refractory skin inflammation in a child with STAT1 GOF disease, highlighting its potential in managing localized inflammation in immune dysregulation.

Heterozygous germline gain-of-function (GOF) mutations in the *STAT1* gene, first identified in 2011, are the main cause of autosomal dominant chronic mucocutaneous candidiasis (CMC). Over 400 cases involving 116 mutations have been reported worldwide. These mutations impair interleukin (IL)-17–mediated immunity, disrupting T helper type 1 cells (Th1) and T helper type 17 cells (Th17) development, leading to Th17 deficiency and impaired fungal immunity, particularly to *Candida* species (1, 2). In addition, signal transducer and activator of transcription 1 GOF (STAT1 GOF) mutations drive hyperactivation of STAT1 signaling in response to type I and II interferons (IFNs) and IL-27, contributing to an overall enhancement of their downstream signaling pathways. Dysregulated IFN signaling plays a key role in the pathogenesis of inflammatory disease and autoimmunity (2).

In fact, although CMC is the hallmark of STAT1 GOF syndrome, its presentation varies extensively, including systemic infections, autoimmune/autoinflammatory conditions, vascular malformations, and malignancies (2). Dermatological manifestations comprise CMC, dermatophytosis, herpes simplex and zoster, and rosacea-like demodicosis, although the exact pathogenesis of demodicosis in STAT1 GOF patients remains unclear. *Demodex* mites overgrowth, often associated with immunodeficiency, triggers toll-like receptor 2–mediated inflammation, causing papulopustular facial lesions. Demodicosis is managed with topical agents like permethrin, ivermectin, or metronidazole, while severe cases may require systemic therapies (3).

In STAT1 GOF syndrome, dealing with treatment-resistant infections and chronic skin disorders remains challenging. Azole-resistant CMC frequently require systemic antimycotics, involving significant toxicity. Hematopoietic stem cell transplantation (HSCT) offers a potential cure, but its failure rates are substantial (2).

Recent advances in understanding the pathophysiology of STAT1 GOF disease highlight the Janus kinase (JAK)-STAT pathway as a therapeutic target. Janus-associated kinase inhibitors (JAKi) restore immune balance by normalizing Th1 and follicular Th populations, reverting IL-17A production, and enhancing natural killer cell function. Nevertheless, systemic JAKi entail risks, in particular infections, including reactivation of herpes zoster and bone marrow suppression (4). To alleviate these concerns, topical JAKi offer targeted and localized immune modulation with fewer systemic effects (5). Ruxolitinib, a selective inhibitor of JAK1 and JAK2, is Food and Drug Administration-approved as a 1.5% cream (Opzelura) for atopic dermatitis (2021) and nonsegmental vitiligo (2022) and has proven efficacy and safety in reducing localized inflammation.

We report the first use of topical JAKi to address a refractory facial rash in a STAT1 GOF patient, providing a novel therapeutic approach for complex skin manifestations in this condition. Written consent was given by family to publish this case report.

A 10-year-old girl, born at full-term after an uncomplicated pregnancy, presented at 13 mo of age with severe CMC involving the oral and perianal areas, since her first month of life. Despite multiple courses of oral and topical antifungal therapies, CMC was unresponsive, causing poor feeding and growth on the second percentile. Her father reported recurrent CMC since his childhood, but a genetic diagnosis was not established at the time.

**Figure 1.**
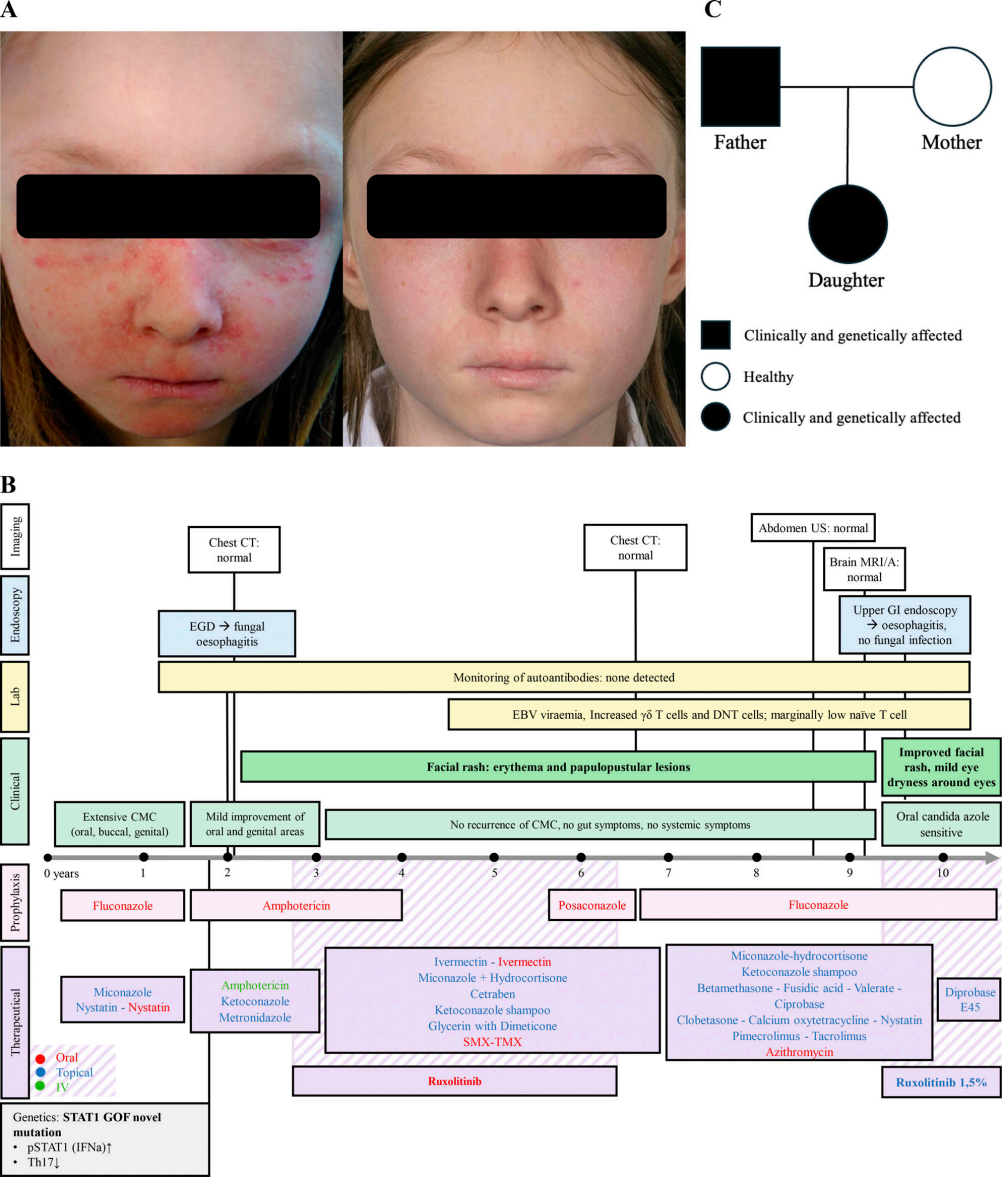
**Clinical summary of patient including evolution of facial rash, clinical timeline, and pedigree. (A)** Progressive improvement of facial erythema and papulopustular lesions observed over time before and after initiating topical ruxolitinib in a 10-year-old patient with STAT1 GOF syndrome. **(B)** Timeline summarizing the patient’s clinical and treatments course. Green: IV treatments; red: oral treatments; blue: topical treatments. **(C)** Pedigree illustrating the inheritance of the STAT1 variant c.974T>A (p.Met325Lys). The father (black square) is clinically and genetically affected, carrying the heterozygous variant and showing clinical features of CMC. The mother (white circle) is healthy and does not carry the variant. The daughter (black circle) is both clinically affected and heterozygous for the same STAT1 variant. EBV, Ebstein-Barr virus; DNT, double-negative T cells; INFa, INF α; IV, intravenous; SMX-TMX, sulfamethoxazole-trimethoprim; MRI/A, magnetic resonance imaging/angiography; US, ultrasound; CT, computed tomography; EGD, esophagogastroduodenoscopy.

Th17 immunity showed nearly absent CD4^+^Ro^+^IL-17^+^ T cells (0.1%), consistent with Th17 cell deficiency. Subsequent genetic testing identified a novel *STAT1* variant (c.974T>A p.Met325Lys, within the DNA-binding domain), which was also identified in her father (Fig. 1 C). Together with *STAT1* hyperphosphorylation in response to IFN-α stimulation, these findings were consistent with STAT1 GOF disease.

At 17 mo, oral amphotericin B prophylaxis was started for pan-azole-resistant CMC. By the age of 2 years, fungal esophagitis revealed by endoscopy necessitated intravenous liposomal amphotericin B, but subsequent switches to oral prophylaxis led to CMC recurrences. Consequently, systemic ruxolitinib (JAKi) was initiated, significantly improving her clinical phenotype. Over 3.5 years on JAKi, antifungal prophylaxis was discontinued due to potential drug interactions, with no CMC recurrence. After stopping ruxolitinib at the age of 6 years, antifungal prophylaxis was reinstated, maintaining CMC control and constant weight gain.

At 2 years of age, she developed a persistent erythematous papulonodular facial rash. Biopsy revealed folliculitis and presence of *Demodex* mites. Despite effective CMC control over time, the rash persisted and flared intermittently, impairing her quality of life and social interactions. Extensive treatments, including oral and topical antibacterial (e.g., azithromycin, metronidazole), antifungal (e.g., ketoconazole, miconazole), antiparasitic (e.g., ivermectin), potent topical corticosteroids (e.g., betamethasone, hydrocortisone), and calcineurin inhibitors (e.g., tacrolimus), failed to resolve the rash.

At the age of 9, following approval from the hospital’s Drugs and Therapeutics Committee, compassionate-use topical ruxolitinib (1.5% cream, twice daily) was initiated to address the persistent inflammatory rash. Significant improvement was observed within a few days, and, by 3 mo, her skin was markedly improved, with clear face, ears, mouth, cheeks, and only slight dryness around the eyes (Fig. 1 A).

Currently she remains on fluconazole prophylaxis and topical JAKi. Immunological profile is largely unremarkable, except for marginally reduced naïve CD4/CD8 T cells, intermittently increased γδT cells, elevated double-negative T cells, and intermittent low-level Ebstein-Barr virus viremia. Regular monitoring for immune dysregulation markers and complications of STAT1 GOF syndrome is ongoing. So far, no signs of autoimmunity, recurrent infections, or extra-cutaneous features have been observed. Dermatological follow-up ensures clinical response and safety of topical JAKi over time. A timeline summarizing the clinical and treatment courses is presented in Fig. 1 B.

This case describes the first use of topical JAKi to treat refractory dermatological manifestations in STAT1 GOF syndrome, a rare immunodeficiency marked by chronic inflammation and immune dysregulation. The patient’s persistent facial rash, resistant to conventional therapies, exemplifies the challenges in the control of localized inflammatory manifestations in this disease and the need for targeted interventions. *STAT1* GOF mutations result in the hyperactivation of type I, II, and III IFN pathways, causing immune dysregulation and chronic inflammation (1). These processes create a favorable setting for localized inflammatory responses, as evidenced by the patient’s rash. A skin biopsy revealed the presence of *Demodex* mites, which may have augmented the inflammation, although its exact contribution remains undefined. Considering the localized nature of the condition and risk of systemic immunosuppression, JAKi topical therapy was chosen to modulate the JAK-STAT pathway at the inflammation site (5). Topical ruxolitinib rapidly and lastingly improved the rash with only slight residual skin dryness. In addition to the relief of physical symptoms, the patient’s quality of life significantly improved by reducing the psychological discomfort associated with a chronic visible rash.

Although systemic JAK inhibitors and antifungals, together with HSCT, remain critical for the management of STAT1 GOF disease, this case proves the feasibility of a topical treatment.

As a single case, these findings are not generalizable and highlight the need for further studies to validate topical JAKi’s efficacy in this disease and address long-term safety issues. Future research may also evaluate the wider applicability of topical JAKi in the treatment of skin conditions associated with immune dysregulation.

This report presents topical ruxolitinib as a novel and effective treatment for refractory skin manifestations in STAT1 GOF syndrome, emphasizing the potential of targeted topical immunomodulators in chronic inflammatory skin conditions in the context of immune disorders.
